# Radial access during percutaneous interventions in patients with acute coronary syndromes: should we routinely monitor radial artery patency by ultrasonography promptly after the procedure and in long-term observation?

**DOI:** 10.1007/s10554-014-0518-5

**Published:** 2014-08-22

**Authors:** Anna Lisowska, Małgorzata Knapp, Agnieszka Tycińska, Piotr Sielatycki, Robert Sawicki, Paweł Kralisz, Włodzimierz J. Musiał

**Affiliations:** 1 Departament of Cardiology, Medical University of Bialystok, Ul. Skłodowskiej 24A, 15-276 Białystok, Poland; 2 Department of Invasive Cardiology, Medical University of Bialystok, Ul. Skłodowskiej 24A, 15-276 Białystok, Poland

**Keywords:** Radial access, Radial artery occlusion, Ultrasonography

## Abstract

Access-site vascular complications in patients undergoing transradial coronary procedures are rare but may have relevant clinical consequences. The aim of the study was to evaluate: (1) radial artery’s (RA) patency immediately after the procedure and in long-term observation, (2) factors influencing the frequency of radial artery’s occlusion (RAO) after percutaneous coronary intervention (PCI) procedures performed via transradial access in the group of 220 patients with acute coronary syndromes (ACS). RA ultrasound was performed 48–72 h after the procedure and in those who were diagnosed with RAO-again after 6–12 months. According to the ultrasonographic findings, the patients were divided into two sub-groups: 187 pts (85 %) with patent RA after PCI and 33 pts (15 %) with RAO. Both sub-groups significantly statistically differed with regard to the frequency of local hematomas—15 versus 27.3 % (*p* = 0.02), the frequency of applying IIbIIIa inhibitors in PCI—6.4 versus 15.1 % (*p* = 0.015) and procedure duration—0.59 ± 0.37 versus 0.77 ± 0.38 (*p* = 0.014), respectively. In a multifactorial analysis the only factor influencing RA patency promptly after the procedure was PCI duration (*p* < 0.05, r = −0.22). In the follow-up, right RA remained still obstructed in 28 patients (12.7 %) whereas in five patients (2.3 %) the regular flow in RA was resumed. The chronic RAO was clinically silent. Due to insignificant frequency of the occurrence of RAO after PCI procedure in patients with ACS as well as practically lack of clinical consequences of this artery’s occlusion in long-term observation, we do not see any implications to routine ultrasound periprocedural RA evaluation.

## Introduction

The entry site complications associated with artery cannulation during coronary angiographies and percutaneous coronary interventions (PCIs) delay discharge and require additional procedures. Radial and femoral approaches are both safe and effective for PCI. However, the lower rate of local vascular complications may be a reason to use the radial approach [1]. The transradial approach for percutaneous coronary intervention and angiography has been shown to be safe and effective alternative to the traditional transfemoral approach. Transradial PCI has been shown to reduce access site-related bleeding complications compared with procedures performed through a femoral approach. Used extensively throughout Europe for the past 15 years, this technique has been recently recommended accordingly to ESC guidelines. Lower direct costs, fewer vascular complications, better patient acceptance and earlier ambulation are some of the direct benefits from using radial access [2]. Access-site vascular complications in patients undergoing transradial coronary procedures are rare but may have relevant clinical consequences [3]. These can be: spasm, occlusion or perforation of radial artery (RA), hematoma, pseudoaneurysm, arteriovenous fistula, nerve injury [4]. The most common complication is asymptomatic RA occlusion, which rarely leads to clinical events, owing to the dual collateral perfusion of the hand. RA spasm is relatively common and can result in access and procedural failure. RA perforation can lead to severe forearm hematoma and compartment syndrome. Pseudoaneurym and arteriovenous fistula are rare complications, which can likely be managed conservatively without surgical intervention. Nerve injury occurring during access has been reported, although symptoms usually improve over time [4].

The aim of the study was to evaluate: (1) RA’s patency immediately after the procedure and in long-term observation as well as the consequences of RA’s chronic obstruction, (2) factors influencing the frequency of RA obstruction after PCI procedures performed via transradial access in the group of patients suffering from acute coronary syndromes (ACS).

## Methods

### Selection of the study population

The study comprised consecutive patients with ACS (STEMI and NSTEMI) who were hospitalized in Department of Cardiology in years 2010–2012 and underwent invasive procedures: coronary angiography and angioplasty via RA access. The inclusion criteria were: age between 18 and 80 years, a completed coronary angiography and doppler ultrasound examination of the RA. Selected clinical and biochemical risk factors of atherosclerosis progression were assessed. Following the above criteria, 220 patients were enrolled. According to the ultrasonographic findings, patients were divided into two sub-groups: 187 pts with patent RA after PCI and 33 pts with occluded RA after PCI. Detailed clinical and procedural characteristics of these groups are given in Tables 1 and 2.

**Table 1:** Clinical characteristic of study group

	Whole study group (n = 220)	Patients with patent radial artery after PCI (n = 187)	Patients with occluded radial artery after PCI (n = 33)	p (pts with patent versus occluded radial artery)
Age (y)	64.0 ± 12.2	64.0 ± 11.9	63.4 ± 13.5	NS
Men	167 (75.6 %)	147 (78.6 %)	20 (60.6 %)	p = 0.025
STEMI	133 (60.2 %)	110 (58.8 %)	17 (51.5 %)	NS
NSTEMI	88 (39.8 %)	71 (38.0 %)	14 (42.4 %)	NS
EF (%)	46.7 ± 9.7	46.9 ± 9.2	45.7 ± 12.4	NS
Creatinine (µmol/l)	122.8 ± 21.0	130.8 ± 53.0	76.9 ± 21.2	*p* = 0.04
Total cholesterol (mmol/l)	4.7 ± 1.1	4.6 ± 1.1	5.0 ± 1.25	NS
LDL-cholesterol (mmol/l)	3.0 ± 1.0	2.9 ± 1.0	3.3 ± 1.1	NS
HDL-cholesterol (mmol/l)	1.1 ± 0.3	1.1 ± 0.35	1.1 ± 0.3	NS
Glucose(mmol/l)	6.3 ± 1.8	6.4 ± 1.8	6.2 ± 1.2	NS

**Table 2:** Procedural characteristic of study group

	Whole study group (n = 220)	Patients with patent radial artery after PCI (n = 187)	Patients with occluded radial artery after PCI (n = 33)	*p* (pts with patent versus occluded radial artery)
Angiographically				
1-vessel disease	106 (47.9 %)	94 (50.3 %)	15 (45.5 %)	NS
2-vessel disease	63 (28.5 %)	55 (29.4 %)	11 (33.3 %)	
3-vessel disease	45 (20.4 %)	38 (20.3 %)	7 (21.2 %)	
PCI LAD	100 (45.5 %)	83 (44.4 %)	15 (45.4 %)	NS
PCI RCA	68 (30.9 %)	56 (30.0 %)	12 (36.3 %)	NS
PCI Cx	52 (23.6 %)	48 (25.7 %)	6 (18.2 %)	NS
Stent (number of pts)	204 (92.7 %)	173 (92.5 %)	31 (93.9 %)	NS
Number of patients with				
1 stent	149 (73.0 %)	125 (72.5 %)	24 (77.4 %)	NS
2 stents	51 (25 %)	45 (26.0 %)	6 (19.4 %)	
3 stents	4 (2.0 %)	3 (1.7 %)	1 (3.2 %)	
BMS (number of pts)	167 (81.8 %)	143 (82.6 %)	24 (77.4 %)	NS
DES (number of pts)	37 (18.1 %)	30 (17.3 %)	7 (22.6 %)	NS
Flow velocity in the right radial artery (cm/s)		60.7 ± 19.2	0	
Flow velocity in the left radial artery (cm/s)	62.8 ± 19.2	63.0 ± 17.4	62.1 ± 27.7	NS
Local hematoma		28 (15.0 %)	9 (27.3 %)	*p* = 0.02
Dose of heparin during PCI	5.85 ± 2.25	5.88 ± 2.26	5.64 ± 2,18	NS
Blocker GP IIbIIIa during PCI	17 (7.7 %)	12 (6.4 %)	5 (15.1 %)	*p* = 0.015
PCI duration (hours)	0.62 ± 0.37	0.59 ± 0.37	0.77 ± 0.38	*p* = 0.014
Previously PCI via radial artery	13 (5.9 %)	11 (5.9 %)	2 (6.1 %)	NS

### Coronary angiography

PCIs were performed in the course of ACS. During coronary angiography 6F catheter was used. PCIs were routinely performed via right RA. The patients who underwent the procedure via femoral artery for technical reasons were excluded from the study. Before the procedure, all the patients had performed Allen test and received standard antiplatelet treatment (acetylsalicylic acid and clopidogrel) in loading doses. As a rule, RA puncture and vascular sheath insertion are followed by injection of verapamil (2.5 mg diluted in 2 ml of 0.9 % NaCl) and 5,000 U unfractionated heparin (UFH). During PCI ACT was controlled (300 s) and additional UFH was added if needed. Platelet glycoprotein IIb/IIIa receptor antagonists were used according to the operator`s decision. After the procedure and vascular sheath removal, as a rule, compression with a tourniquet (SUNMED™ Disposable TR—Closure Band, As Medical, Netherlands) was applied followed by 4–6-h. Experienced operators were highly experimented in radial access (>80 % of their procedures routinely performed by radial access during the past 2 years) and high volume operators (>100 procedures per year).

### Doppler ultrasonography of the radial arteries

Radial artery ultrasound was routinely performed in the second or third day after the procedure, independently on the access site complcations. Philips iE 33 ultrasound equipment with a 3–11 MHz linear transducer was used. Blood flow was assessed with colour-Doppler sonography in 2D images. The flow in both right and left RA was studied. The nonintervened left RA served as the control. Next in the group of patients who were diagnosed with periprocedural right RA obstruction the ultrasound control test was performed again after the lapse of app. 6–12 months.

### Statistical analysis

The mean values and standard deviations for quantitative variables were calculated as well as the quantitative and percentage distribution for qualitative variables. Pearson’s correlation coefficient was calculated for categorical variables of normal distribution and Spearman’s correlation coefficient for variables not satisfying normal distribution criteria. To compare the groups, the statistical analysis for variables of normal distribution estimated using the Kołomogorow compatibility test was carried out using the unpaired Student’s test and the Mann–Whitney test for variables inconsistent with a normal distribution. A comparison of qualitative variables between the groups was performed using the χ^2^ test. A *p* value of <0.05 was considered statistically significant.

The statistical analysis was carried out using Statistica 10.0 PL software.

## Results

Detailed characteristics of the studied group is presented in Tables 1 and 2. According to the ultrasonographic findings, the patients were divided into two sub-groups: 187 pts (85 %) with patent RA after PCI and 33 pts (15 %) with occluded RA after PCI. The patients in both groups did not differ significantly with regard to age, the frequency of STEMI/NSTEMI occurrence, concentration of total, LDL and HDL cholesterol and glucose or a magnitude of ejection fraction assessed echocardiographically. In the group of patients with occluded RA, creatinine concentration was lower, and more often these were female patients.

As far as procedural and periprocedural characteristics of the studied group is concerned, there were no statistically significant differences between the sub-groups in the scope of: a degree of advancement of atherosclerotic changes in coronary arteries, the localization of the lesions, a number of implanted stents, a type of applied stent (BMS vs DES), a dose of heparin applied in the procedure, and a number of previously performed PCI via transradial approach. On the other hand, both sub-groups significantly statistically differed with regard to the frequency of the occurrence of local hematomas—27.3 % in pts with occluded RA versus 15.0 % in pts with patent RA after PCI (*p* = 0.02), the frequency of applying IIbIIIa inhibitors during PCI—15.1 % versus 6.4 % (*p* = 0.015) and procedure duration—0.77 ± 0.38 versus 0.59 ± 0.37 (*p* = 0.014), respectively.

None of the patients required local surgical intervention. Conservative management including local compression allows successful management in all cases of local complications. After the performance of a multifactorial analysis, it appeared that the only factor influencing RA patency promptly after the procedure is PCI duration (*p* < 0.05, r = −0.22).

### Follow-up

The follow-up period amounted to 6–12 months. In 33 (15 % of whole group) patients who were diagnosed with periprocedural obstruction of right RA, after the follow-up period the right RA remained still obstructed in 28 patients (12.7 %) whereas in 5 patients (2.3 %) a regular flow in the RA was resumed. In the group of patients with persistent obstruction of RA, only one patient exhibited a mild neurological hand deficit in the follow-up (right arm’s coldness, periodic paraesthesia). The resumption of RA patency correlated statistically significantly with PCI procedure duration (*p* < 0.05, *r* = −0.17).

## Discussion

Transradial approach to coronary angiography is a progressive and increasingly more often used technique. RA access is associated with lower risk of any complications or access site bleeding complications. Use of the RA for primary or rescue PCI is associated with improved clinical outcomes [5]. Apart from its advantages, it is important to remember four important issues related to transradial procedures: (1) radial access site bleeding, (2) RA injury and occlusion, (3) radiation exposure, and (4) implementation of a successful transradial primary percutaneous coronary intervention [6]. The most common complication is RA occlusion, which rarely leads to clinical events, owing to the dual collateral perfusion of the hand. In our own studies performed straight after the procedure, RA obstruction was found in 15 % patients, which was asymptomatic in almost all patients, i.e. except one patient. RA occlusion (RAO) represents the most serious drawback—usual with an incidence of 4–12 % in the literature [7–10]. Slightly higher incidence of early RAO in our study may be due to the fact that in all cases, PCI were performed in the course of ACS, these procedures were not planned. Moreover, in the most studies cited above, the presence of flow in the RA after the procedure was evaluated clinically [7–9], and in just a few [10]—ultrasound examination was performed, as in our population. Re-canalization of an iatrogenic RAO, although asymptomatic in the majority of cases, remains a discussed and challenging topic [11]. It is postulated that appropriate compression techniques and smaller sheath size can minimize the risk of RA occlusion [4]. In our centre during angiography 6F catheter was applied. After the procedure and vascular sheath removal, as a rule, compression with a tourniquet was applied followed by 4–6-h. During the follow–up in the studied group of patients RA remained still obstructed in 12.7 % patients whereas in 2.3 % patients a regular flow in RA was resumed. In the group of patients suffering from persistent RA obstruction only one patient exhibited a mild neurological hand deficit in the follow-up. The large proportion of late occlusion of the RA during the follow-up period of 6–12 months observed in our study population differs from the data available in the literature. Other researchers have observed a return flow in the initially occluded RA after 1 month at an average of 30–40 % of patients [12, 13], and in our group of patients, this percentage was only 15 %. According to the authors’ knowledge, there is no data concerning longer follow-up.

In our study, (after performance of a multifactorial analysis) it appeared that the only factor influencing RA patency promptly after the procedure was PCI duration. Hand ischemia with necrosis has never been reported in our study group, which is consistent with other authors’ observations [4]. Although the RA is superficial and haemostasis can be achieved readily, access site bleeding can occur and can lead to forearm haematoma and, rarely, to compartment syndrome, if not managed promptly. In our study local hematomas occurred significantly more frequenlty in patients suffering from obstructed RA after the procedure. Nonetheless, no patient required local surgical intervention. Conservative management including local compression allows successful management in all cases of local complications. Pseudoaneurym and arteriovenous fistula are rare complications. Moreover, we found a case report of right RA perforation observed after successful stenting of left anterior descending artery through right radial access [14, 15]. In the group of our patients we did not observe such type of complications.

The potential reduction in mortality seen with transradial primary PCI must be balanced against the clinical imperative of timely reperfusion. Operators and catheterization laboratories should not begin a transradial primary PCI programme until sufficient radial experience has been gained in the elective setting [6]. In our department PCIs were performed by experienced operators (highly experimented in radial access and high volume operators).

In the studied group of patients with occluded RA after PCI creatinine concentration was lower, and more often these were female patients. Other studied clinical and biochemical parametres appeared insignificant. It appeared in other authors’ studies that age, sheath size, the dose of heparin were significantly higher in patients with than without bleeding complications. However, body mass index (BMI) was significantly lower in patients with than without bleeding complications [16]. Sheath size was significantly higher in patients with than without RA occlusion [16]. In our study during coronary angiography 6F catheter was used in all patients. Multiple logistic analysis revealed that the only factor influencing RA patency promptly after the procedure was its duration.

Right and left radial approaches are feasible and effective to perform percutaneous procedures. However, the left radial route is associated with shorter procedures and lower radiologic exposure than the right radial approach, independently of an operator’s proficiency [17]. In our study PCIs were routinely performed via right RA. Left RA was left intact with a view of a possible use of this artery as a bridge in case the patient qualifies for Coronary Artery Bypass Graft procedure in the future. We should remember that a marked narrowing in the diameter of the intervened RA and impaired FMD response indicating endothelial dysfunction were observed at a mean of 9 months after transradial intervention. Structural and functional changes should be taken into consideration if previously intervened RA would be used for interventions, such as arterial bypass graft or dialysis fistula [18].

In our study group there were no significant differences in spite of a higher incidence of radial artery occlusion (RAO) in patients with previously performed PCI via transradial approach. Moreover, similar results were obtained by other researchers [19].

A sterile inflammatory reaction at the radial access site has been described in the literature as an adverse local reaction to Cook hydrophilic coated sheaths during transradial catheterization. Until now, this reaction has not been observed with non-Cook hydrophilic sheaths [20]. In our cathlab non-Cook hydrophilic sheaths are applied—we did not observe any allergic reactions as well.

### Study limitations

The main study limitation is small size of the study group.

## Conclusions

Due to insignificant frequency of the occurrence of RA occlusion after PCI procedure in patients with ACS as well as practically lack of clinical consequences of this artery’s occlusion in long-term observation, we do not see any implications to routine ultrasound periprocedural RA evaluation. Control ultrasonographic tests should be reserved solely to patients with a clinical picture that may suggest the incidence of local complications. The only factor influencing RA patency promptly after the procedure was PCI duration. Moreover, ultrasonographic and clinical control after 6–12 months after the procedure is not necessary because persisting obstruction of RA does not generate symptoms.
